# 2-Chloro-8-methyl­quinoline-3-carbaldehyde

**DOI:** 10.1107/S1600536809040859

**Published:** 2009-10-13

**Authors:** F. Nawaz Khan, R. Subashini, Atul Kumar Kushwaha, Venkatesha R. Hathwar, Seik Weng Ng

**Affiliations:** a Chemistry Division, School of Science and Humanities, VIT University, Vellore 632 014, Tamil Nadu, India; b Solid State and Structural Chemistry Unit, Indian Institute of Science, Bangalore 560 012, Karnataka, India; c Department of Chemistry, University of Malaya, 50603 Kuala Lumpur, Malaysia

## Abstract

The quinoline fused-ring system of the title compound, C_11_H_8_ClNO, is planar (r.m.s. deviation = 0.005 Å); the formyl group is slightly bent out of the plane [C—C—C–O1 torsion angles = 8.8 (7) and −172.8 (4)°].

## Related literature

For a review of the synthesis of quinolines by the Vilsmeier–Haack reaction, see: Meth-Cohn (1993 ▶).
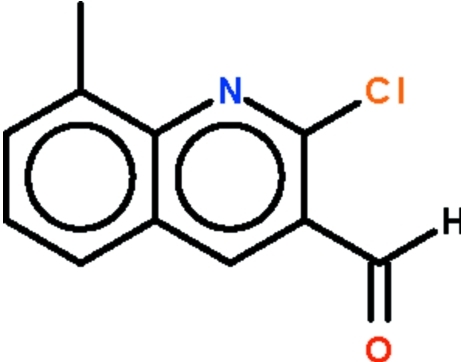


## Experimental

### 

#### Crystal data

C_11_H_8_ClNO
*M* *_r_* = 205.63
Orthorhombic, 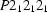

*a* = 6.8576 (5) Å
*b* = 7.4936 (6) Å
*c* = 18.5003 (14) Å
*V* = 950.70 (13) Å^3^
*Z* = 4
Mo *K*α radiation
μ = 0.36 mm^−1^
*T* = 290 K
0.26 × 0.22 × 0.17 mm

#### Data collection

Bruker SMART area-detector diffractometer
Absorption correction: multi-scan (*SADABS*; Sheldrick, 1996 ▶) *T*_min_ = 0.912, *T*_max_ = 0.941
8224 measured reflections
2174 independent reflections
1734 reflections with *I* > 2σ(*I*)
*R*_int_ = 0.043

#### Refinement

*R*[*F* ^2^ > 2σ(*F* ^2^)] = 0.048
*wR*(*F* ^2^) = 0.136
*S* = 1.00
2174 reflections
129 parameters
H-atom parameters constrained
Δρ_max_ = 0.25 e Å^−3^
Δρ_min_ = −0.33 e Å^−3^
Absolute structure: Flack (1983 ▶), 838 Friedel pairs
Flack parameter: 0.2 (2)

### 

Data collection: *SMART* (Bruker, 2004 ▶); cell refinement: *SAINT* (Bruker, 2004 ▶); data reduction: *SAINT*; program(s) used to solve structure: *SHELXS97* (Sheldrick, 2008 ▶); program(s) used to refine structure: *SHELXL97* (Sheldrick, 2008 ▶); molecular graphics: *X-SEED* (Barbour, 2001 ▶); software used to prepare material for publication: *publCIF* (Westrip, 2009 ▶).

## Supplementary Material

Crystal structure: contains datablocks global, I. DOI: 10.1107/S1600536809040859/bt5085sup1.cif

Structure factors: contains datablocks I. DOI: 10.1107/S1600536809040859/bt5085Isup2.hkl

Additional supplementary materials:  crystallographic information; 3D view; checkCIF report

## Figures and Tables

**Fig. 1.**
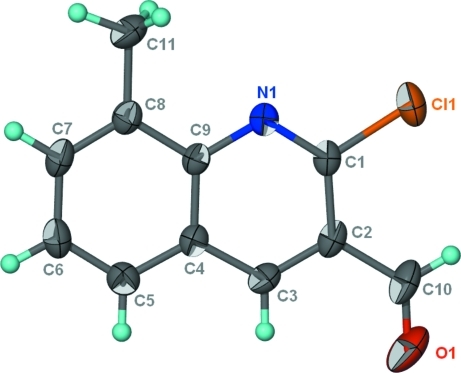
Thermal ellipsoid plot (Barbour, 2001) of C11H8ClNO at the 50% probability level; hydrogen atoms are drawn as spheres of arbitrary radius.

## supplementary crystallographic information

### Experimental

The Vilsmeier-Haack reagent prepared from phosphorus oxytrichloride (6.5 ml, 70 mmol) and *N*,*N*-dimethylformamide (2.3 ml, 30 mmol) at 273 K was added *N*-(2-tolyl)acetamide (1.49 g, 10 mmol). The mixture was heated at 353 K for 15 h. The mixture was poured onto ice; the white product was collected and dried. The compound was purified by recrystallization from a petroleum ether/ethyl acetate mixture.

### Refinement

Carbon-bound H-atoms were placed in calculated positions (C–H 0.93–0.96 Å) and were included in the refinement in the riding model approximation, with *U*(H) set to 1.2–1.5*U*(C).

The crystal had two domains related by a translation of (1/2, 0, 0) so that all reflections with *h* = 2*n* are affected. A scale factor was added for all reflections with *h* = 2*n*. The *hkl* file had a scale factor of 1 for *h *= 2*n* + 1 and a scale factor of 2 for the *h* = 2*n* reflections.

### Crystal data

**Table:**

C_11_H_8_ClNO	*F*(000) = 424
*M**_r_* = 205.63	*D*_x_ = 1.437 Mg m^−^^3^
Orthorhombic, *P*2_1_2_1_2_1_	Mo *K*α radiation, λ = 0.71073 Å
Hall symbol: P 2ac 2ab	Cell parameters from 867 reflections
*a* = 6.8576 (5) Å	θ = 2.0–24.4°
*b* = 7.4936 (6) Å	µ = 0.36 mm^−^^1^
*c* = 18.5003 (14) Å	*T* = 290 K
*V* = 950.70 (13) Å^3^	Block, colorless
*Z* = 4	0.26 × 0.22 × 0.17 mm

### Data collection

**Table:**

Bruker SMART area-detector diffractometer	2174 independent reflections
Radiation source: fine-focus sealed tube	1734 reflections with *I* > 2σ(*I*)
graphite	*R*_int_ = 0.043
φ and ω scans	θ_max_ = 27.5°, θ_min_ = 2.2°
Absorption correction: multi-scan (*SADABS*; Sheldrick, 1996)	*h* = −8→8
*T*_min_ = 0.912, *T*_max_ = 0.941	*k* = −9→9
8224 measured reflections	*l* = −22→24

### Refinement

**Table:**

Refinement on *F*^2^	Secondary atom site location: difference Fourier map
Least-squares matrix: full	Hydrogen site location: inferred from neighbouring sites
*R*[*F*^2^ > 2σ(*F*^2^)] = 0.048	H-atom parameters constrained
*wR*(*F*^2^) = 0.136	*w* = 1/[σ^2^(*F*_o_^2^) + (0.0861*P*)^2^ + 0.0263*P*] where *P* = (*F*_o_^2^ + 2*F*_c_^2^)/3
*S* = 1.00	(Δ/σ)_max_ = 0.001
2174 reflections	Δρ_max_ = 0.25 e Å^−^^3^
129 parameters	Δρ_min_ = −0.32 e Å^−^^3^
0 restraints	Absolute structure: Flack (1983), 838 Friedel pairs
Primary atom site location: structure-invariant direct methods	Flack parameter: 0.2 (2)

### Fractional atomic coordinates and isotropic or equivalent isotropic displacement parameters (Å^2^)

**Table:**

	*x*	*y*	*z*	*U*_iso_*/*U*_eq_	
Cl1	0.87010 (13)	1.14505 (9)	0.37890 (4)	0.0579 (3)	
O1	0.8672 (5)	0.6106 (3)	0.29577 (10)	0.0763 (6)	
N1	0.8717 (4)	0.9871 (2)	0.50346 (10)	0.0366 (4)	
C1	0.8734 (5)	0.9563 (3)	0.43459 (13)	0.0368 (5)	
C2	0.8754 (5)	0.7858 (3)	0.40176 (11)	0.0398 (5)	
C3	0.8734 (5)	0.6423 (4)	0.44734 (12)	0.0389 (5)	
H3	0.8741	0.5276	0.4281	0.047*	
C4	0.8705 (4)	0.6651 (3)	0.52293 (10)	0.0344 (5)	
C5	0.8691 (5)	0.5212 (3)	0.57186 (14)	0.0443 (6)	
H5	0.8707	0.4045	0.5547	0.053*	
C6	0.8654 (6)	0.5531 (3)	0.64410 (13)	0.0468 (6)	
H6	0.8633	0.4585	0.6766	0.056*	
C7	0.8648 (5)	0.7301 (4)	0.66955 (13)	0.0443 (6)	
H7	0.8614	0.7491	0.7192	0.053*	
C8	0.8690 (4)	0.8754 (3)	0.62492 (11)	0.0372 (5)	
C9	0.8702 (4)	0.8426 (3)	0.54918 (11)	0.0327 (4)	
C10	0.8840 (6)	0.7545 (5)	0.32246 (14)	0.0551 (7)	
H10	0.9034	0.8521	0.2922	0.066*	
C11	0.8698 (7)	1.0624 (3)	0.65349 (14)	0.0547 (7)	
H11A	0.8779	1.0598	0.7053	0.082*	
H11B	0.7519	1.1218	0.6392	0.082*	
H11C	0.9801	1.1255	0.6343	0.082*	

### Atomic displacement parameters (Å^2^)

**Table:**

	*U*^11^	*U*^22^	*U*^33^	*U*^12^	*U*^13^	*U*^23^
Cl1	0.0687 (4)	0.0591 (4)	0.0459 (4)	0.0008 (5)	0.0006 (4)	0.0218 (3)
O1	0.0887 (16)	0.1007 (17)	0.0394 (10)	−0.008 (2)	−0.0031 (13)	−0.0254 (11)
N1	0.0371 (10)	0.0403 (10)	0.0324 (10)	−0.0007 (12)	0.0005 (12)	0.0028 (7)
C1	0.0327 (11)	0.0459 (13)	0.0318 (12)	0.0001 (17)	−0.0007 (16)	0.0062 (10)
C2	0.0347 (11)	0.0576 (14)	0.0272 (10)	−0.0003 (15)	−0.0008 (12)	−0.0019 (10)
C3	0.0393 (11)	0.0427 (11)	0.0348 (12)	−0.002 (2)	−0.0005 (14)	−0.0084 (10)
C4	0.0323 (10)	0.0410 (11)	0.0300 (10)	−0.0007 (15)	0.0005 (11)	−0.0004 (8)
C5	0.0509 (14)	0.0398 (12)	0.0422 (14)	0.0026 (19)	0.0008 (19)	0.0040 (10)
C6	0.0509 (14)	0.0519 (14)	0.0375 (12)	0.0021 (17)	0.0007 (16)	0.0120 (10)
C7	0.0457 (14)	0.0596 (15)	0.0276 (11)	0.0043 (19)	−0.0022 (15)	0.0020 (11)
C8	0.0344 (10)	0.0461 (12)	0.0310 (11)	0.0029 (12)	−0.0009 (13)	−0.0014 (9)
C9	0.0292 (10)	0.0407 (11)	0.0280 (10)	−0.0001 (16)	0.0004 (12)	0.0002 (9)
C10	0.0538 (17)	0.084 (2)	0.0278 (12)	0.000 (2)	−0.0010 (17)	−0.0032 (13)
C11	0.0716 (17)	0.0543 (15)	0.0384 (13)	0.003 (2)	−0.0005 (19)	−0.0128 (11)

### Geometric parameters (Å, °)

**Table:**

Cl1—C1	1.750 (2)	C5—H5	0.9300
O1—C10	1.191 (4)	C6—C7	1.407 (4)
N1—C1	1.295 (3)	C6—H6	0.9300
N1—C9	1.374 (3)	C7—C8	1.367 (3)
C1—C2	1.415 (3)	C7—H7	0.9300
C2—C3	1.367 (3)	C8—C9	1.423 (3)
C2—C10	1.487 (3)	C8—C11	1.498 (3)
C3—C4	1.409 (3)	C10—H10	0.9300
C3—H3	0.9300	C11—H11A	0.9600
C4—C5	1.408 (3)	C11—H11B	0.9600
C4—C9	1.416 (3)	C11—H11C	0.9600
C5—C6	1.358 (4)		
			
C1—N1—C9	117.73 (18)	C8—C7—C6	123.3 (2)
N1—C1—C2	125.68 (19)	C8—C7—H7	118.4
N1—C1—Cl1	115.80 (18)	C6—C7—H7	118.4
C2—C1—Cl1	118.52 (18)	C7—C8—C9	117.2 (2)
C3—C2—C1	116.47 (19)	C7—C8—C11	122.2 (2)
C3—C2—C10	119.0 (2)	C9—C8—C11	120.6 (2)
C1—C2—C10	124.5 (2)	N1—C9—C4	121.95 (19)
C2—C3—C4	121.1 (2)	N1—C9—C8	118.06 (19)
C2—C3—H3	119.4	C4—C9—C8	119.99 (19)
C4—C3—H3	119.4	O1—C10—C2	123.2 (3)
C5—C4—C3	123.0 (2)	O1—C10—H10	118.4
C5—C4—C9	119.9 (2)	C2—C10—H10	118.4
C3—C4—C9	117.0 (2)	C8—C11—H11A	109.5
C6—C5—C4	119.9 (2)	C8—C11—H11B	109.5
C6—C5—H5	120.1	H11A—C11—H11B	109.5
C4—C5—H5	120.1	C8—C11—H11C	109.5
C5—C6—C7	119.7 (2)	H11A—C11—H11C	109.5
C5—C6—H6	120.2	H11B—C11—H11C	109.5
C7—C6—H6	120.2		
			
C9—N1—C1—C2	−0.5 (5)	C6—C7—C8—C9	1.3 (5)
C9—N1—C1—Cl1	178.7 (2)	C6—C7—C8—C11	−179.5 (4)
N1—C1—C2—C3	0.6 (5)	C1—N1—C9—C4	0.0 (4)
Cl1—C1—C2—C3	−178.6 (3)	C1—N1—C9—C8	179.8 (3)
N1—C1—C2—C10	−177.9 (3)	C5—C4—C9—N1	179.9 (3)
Cl1—C1—C2—C10	2.9 (5)	C3—C4—C9—N1	0.3 (5)
C1—C2—C3—C4	−0.3 (5)	C5—C4—C9—C8	0.2 (4)
C10—C2—C3—C4	178.3 (3)	C3—C4—C9—C8	−179.4 (2)
C2—C3—C4—C5	−179.8 (3)	C7—C8—C9—N1	179.1 (3)
C2—C3—C4—C9	−0.1 (5)	C11—C8—C9—N1	−0.1 (5)
C3—C4—C5—C6	−179.7 (4)	C7—C8—C9—C4	−1.1 (4)
C9—C4—C5—C6	0.7 (5)	C11—C8—C9—C4	179.6 (3)
C4—C5—C6—C7	−0.6 (6)	C3—C2—C10—O1	8.8 (7)
C5—C6—C7—C8	−0.4 (6)	C1—C2—C10—O1	−172.8 (4)
